# When sleep disorders in patients with bipolar disorder indicate a risk of suicidal behavior

**DOI:** 10.1192/j.eurpsy.2024.1637

**Published:** 2024-08-27

**Authors:** K. Razki, C. Najar, S. Ben Aissa, M. Oumaya, R. Bouzid

**Affiliations:** ^1^Razi Hospital, LaManouba; ^2^Mental Health Department, Tahar Maamouri Hospital, Nabeul, Tunisia

## Abstract

**Introduction:**

Sleep disturbances and suicidal behaviors are common among patients with type II bipolar disorder ( BDII), but the relationship between the two is unclear. Investigating this connection is important to identify interventions that can improve the quality of life and reduce the risk of suicide in this population.

**Objectives:**

Our study’s objective is to examine the association between sleep disorders and suicidal behavior in patients with type II bipolar disorder (BDII).

**Methods:**

In order to comprehensively investigate the association between sleep disturbances and suicidal behaviors among individuals diagnosed with type II bipolar disorder (TBII), we conducted a cross-sectional, descriptive, and analytical study over a duration of one month, specifically from the 1st to the 31st of October 2022. Our research was conducted within the follow-up unit of the mental health department at Nabeul Hospital, Tunisia, with the aim of capturing a diverse range of participants representative of the population of interest.

To ensure the integrity and accuracy of our findings, we meticulously selected participants who met specific eligibility criteria. This included individuals aged between 18 and 60 years, who had a confirmed diagnosis of type II bipolar disorder according to the criteria outlined in the Diagnostic and Statistical Manual of Mental Disorders (DSM V). Furthermore, we sought to include participants who were psychiatrically stable, meaning they had not required hospitalization in the six months preceding the study.

The Pittsburgh Sleep Quality Index (PSQI) was used to evaluate the participants’ sleep quality over a one-month period, while the Suicidal Behavior Questionnaire-Revised (SBQ-R) was used to assess suicidal behavior.The data was gathered through a questionnaire that prioritized ethical concerns, including obtaining informed consent from participants and maintaining confidentiality and anonymity throughout the study.

**Results:**

In this study, we enrolled 40 male patients with a mean age of 36 ± 13.2 years and evaluated their sleep quality and suicidal behaviors. The results showed that the participants had a mean PSQI score of 7.28 ± 3.35, indicating that the overall sleep quality was not optimal. Specifically, 65% of the participants had poor sleep quality (> 5), and 45% reported poor sleep(PSQI ≥ 8) . The mean SBQ-R score was 10.3 ± 3.6, indicating a moderate level of suicidal behavior. Interestingly, we found a statistically significant correlation between PSQI and SBQ-R subscales, particularly with regard to suicidal thoughts (p=0.003) and suicide attempts (p=0.002).

**Conclusions:**

Our study found a strong link between sleep problems and suicidal behavior in people with type II bipolar disorder. This highlights the need to address sleep issues to reduce suicide risk in these patients.

**Disclosure of Interest:**

None Declared

